# Effect of Ethanol on the Adsorption of Volatile Sulfur Compounds on Solid Phase Micro-Extraction Fiber Coatings and the Implication for Analysis in Wine

**DOI:** 10.3390/molecules24183392

**Published:** 2019-09-18

**Authors:** Peter M. Davis, Michael C. Qian

**Affiliations:** 1 Department of Food Science & Technology, Oregon State University, Corvallis, OR 97331, USA; yungdoufu@gmail.com; 2 Oregon Wine Research Institute, Oregon State University, Corvallis, OR 97331, USA

**Keywords:** volatile sulfur compounds, SPME, wine, GC-PFPD

## Abstract

Complications in the analysis of volatile sulfur compounds (VSC) in wine using solid-phase microextraction (SPME) arise from sample variability. Constituents of the wine matrix, including ethanol, affect the volatility and adsorption of sulfur volatiles on SPME fiber coatings (Carboxen- polydimethylsiloxane(PDMS); DVB-Carboxen-PDMS and DVB-PDMS), which can impact sensitivity and accuracy. Here, several common wine sulfur volatiles, including hydrogen sulfide (H_2_S), methanethiol (MeSH), dimethyl sulfide (DMS), dimethyl disulfide (DMDS), dimethyl trisulfide (DMTS), diethyl disulfide (DEDS), methyl thioacetate (MeSOAc), and ethyl thioacetate (EtSOAc) are analyzed, using SPME followed by gas chromatography (GC), using a system equipped with a pulsed-flame photometric detection (PFPD) system, at various ethanol concentrations in a synthetic wine matrix. Ethyl methyl sulfide (EMS), diethyl sulfide (DES), methyl isopropyl sulfide (MIS), ethyl isopropyl sulfide (EIS), and diisopropyl disulfide (DIDS) are evaluated as internal standards. The absorption of volatile compounds on the SPME fiber is greatly affected by ethanol. All compounds exhibit a stark decrease in detectability with the addition of ethanol, especially between 0.0 and 0.5% *v*/*v*. However, the ratio of interested sulfur compounds to the internal standard becomes more stable when the total alcohol concentration exceeds 2%. EMS was found to best resemble DMS. EIS and DES were found to best resemble DMDS, MeSOAc, and EtSOAc. DIDS was found to best resemble DEDS, DMTS, H_2_S, and MeSH.

## 1. Introduction

Volatile sulfur compounds (VSC), often having very low odor thresholds, are responsible for many common off-flavors in wine [1]. Such compounds often have offensive odors, imparting notes of cabbage, onion, garlic, or rubber to wines [1,2,3,4,5,6]. However, because of their relatively low concentrations, a highly sensitive method is required for accurate analysis.

Solid-phase microextraction (SPME) is a common method for the extraction of volatiles from food and beverage samples [4,7,8]. A coated fiber is extended into the headspace of a sample vial, allowing a finite number of volatiles to adsorb until later thermal desorption. The relatively minute scale of the fiber′s capacity compared to the over sample ensures that the removal of a small fraction of the volatile content will not disturb the sample equilibrium. This also allows for very fast equilibration between the air-fiber interface as compared to static headspace analyses [9]. However, due to the limitation of space for volatiles to adhere to the fiber, the presence of other volatiles from the matrix can interfere with analysis [10].

Ethanol is the major volatile in wine and its concentration often varies greatly among cellars and vintages. This can cause issues with sulfur analysis, as a decrease in analysis sensitivity has been shown in samples with increasing ethanol content [11,12]. While volatile competition on the fiber may be a factor, some have suggested that ethanol acts as a co-solvent in the sample liquid, affecting transition coefficients at the liquid-air interface [13]. This suggestion was a result of ethanol concentration studies using static headspace techniques. Furthermore, it has been shown that varying sample parameters, such as the temperature, extraction time, and matrix can affect individual compounds differently [13]. This is of major concern for wine analysis, as parameters such as ethanol content and volatile and non-volatile profiles are well-varied. For this reason, a proper internal standard must be found for each analyte, ensuring their behaviors in response to parameter variation are consistent and shared.

Carboxen-poly(dimethylsiloxane) (Car/PDMS) is the most commonly-used fiber coating for SPME sulfur analysis due to its high sensitivity. Because of the porous structure of Carboxen particles dispersed in the PDMS phase, it exhibits the displacement effects of other compounds, whereas other more-uniform fibers such as PDMS and DVB/PDMS have no displacement effect [9]. In addition, it has also been shown to have less repeatability [14].

In order to investigate the influence of ethanol on SPME sensitivity, several common sulfur compounds were analyzed on three different fibers. Five internal standards were investigated, including ethyl methyl sulfide (EMS) and diisopropyl disulfide (DIDS). The samples were prepared with identical concentrations of analytes and internal standards, with varying ethanol concentrations.

## 2. Results and Discussion

### 2.1. Headspace-Solid Phase Microextraction (HS-SPME) Analysis

**Figure 1:**
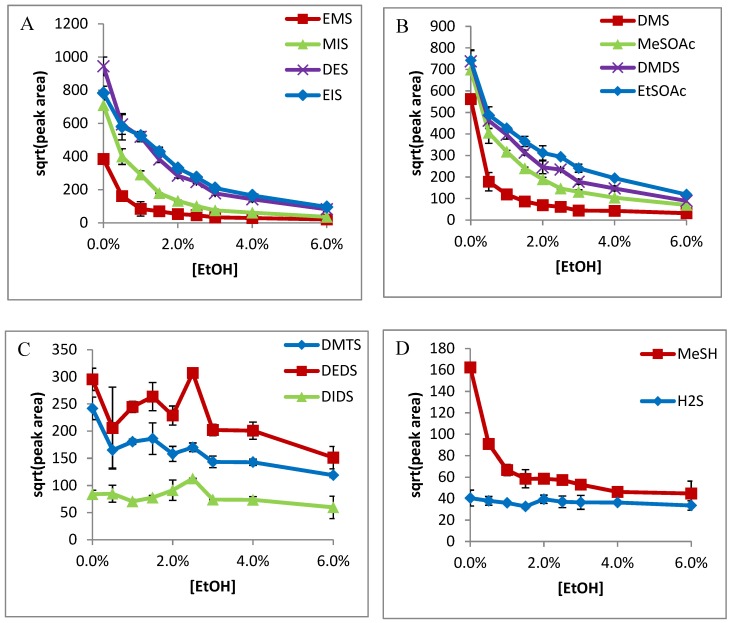
Effects of ethanol on the adsorption of the Carboxen-PDMS solid-phase microextraction (SPME) fiber of: (**A**) ethyl methyl sulfide (EMS), methyl isopropyl sulfide (MIS), diethyl sulfide (DES), and ethyl isopropyl sulfide (EIS); (**B**) dimethyl sulfide (DMS), methyl thioacetate (MeSOAc), dimethyl disulfide (DMDS), and ethyl thioacetate (EtSOAc); (**C**) dimethyl trisulfide (DMTS), diethyl disulfide (DEDS), and diisopropyl disulfide (DIDS); (**D**) methanethiol (MeSH) and H_2_S.

**Figure 2:**
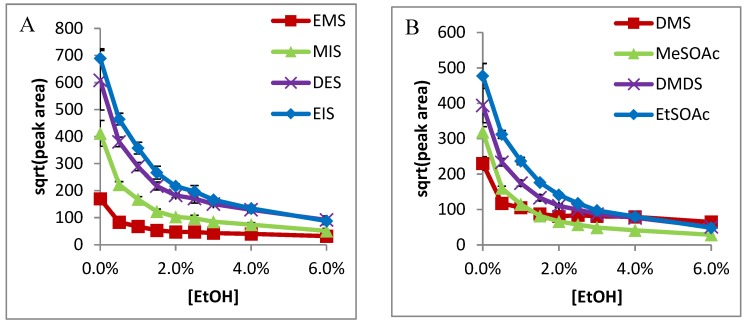

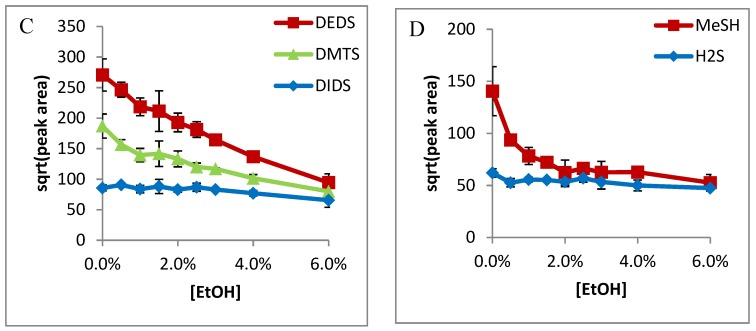
Effects of ethanol on the adsorption of DVB-Carboxen-PDMS SPME fiber of: (**A**) Internal standards EMS, MIS, DES, and EIS; (**B**) DMS, MeSOAc, DMDS, and EtSOAc; (**C**) large compounds DMTS, DEDS, and DIDS (IS); (**D**) highly-volatile compounds MeSH and H_2_S.

**Figure 3:**
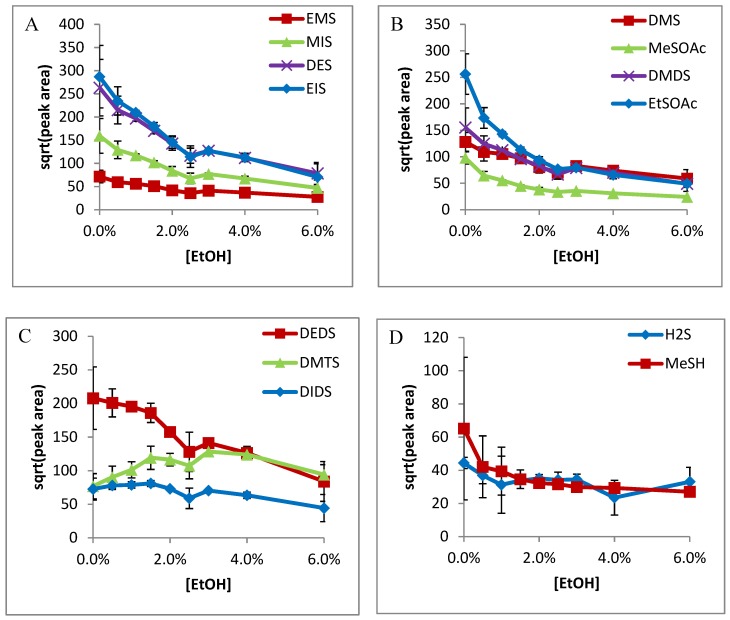
Effects of ethanol on the adsorption of DVB-PDMS SPME fiber of: (**A**) Internals standards EMS, MIS, DES, and EIS; (**B**) DMS, MeSOAc, DMDS, and EtSOAc; (**C**) large compounds DMTS, DEDS, and DIDS (IS); (**D**) highly-volatile compounds MeSH and H_2_S.

In all cases, sensitivity was seen to decrease with increased ethanol content. A very stark decrease was seen immediately with the addition of ethanol (0–0.5%) on Carboxen-PDMS (Figure 1) for smaller compounds, except H_2_S. Ethanol had less influence on the absorption of DMTS, DEDS, and DIDS on DVB/Car/PDMS fiber, and no influence on Car/PDMS fiber. It was postulated that the ethanol competed with adsorption of small VSCs on the active Carboxen site, such that the addition of ethanol could dramatically decrease the absorption of small VSCs on the fiber. On the other hand, H_2_S had a stronger affinity on the Carboxen, so it was not influenced by ethanol content. Similarly, large VSCs including DMTS, DEDS, and DIDS compete more favorably on the Carboxen fiber, so ethanol had no influence on their absorption. Similar trends were observed for DVB-Carboxen-PDMS fibers, except ethanol, which decreased the absorption of DMTS and DEDS (Figure 2). The results from the DVB-PDMS fiber are shown in Figure 3. DMS, DMDS, MeSOAc, EtSOAc (Figure 1B, Figure 2B and Figure 3B), as well as the internal standards EMS, DES, MIS, and EIS (Figure 1A, Figure 2A and Figure 3A), all exhibited similar curve shapes. DEDS, DMTS, and DIDS (Figure 1C, Figure 2C and Figure 3C) shared a different shape, with more gradual decreases with respect to concentration. Methanethiol showed a decrease, but with a more gradual curve, like those of larger species. Hydrogen sulfide exhibits very little influence from ethanol concentration, possibly suggesting the ability to enter very small pores on the Carboxen fiber phase and avoid competition (Figure 1D, Figure 2D and Figure 3D).

The DVB-PDMS fiber showed less sensitivity toward all compounds, but also less dependence on ethanol concentration (Figure 3), as has been previously noted [14]. The smaller compounds and internal standards (such as DIDS) show a less-sudden decrease with ethanol, almost linearly (Figure 3A,B). Larger compounds like DEDS show a gradual decrease, while DMTS and DIDS can be seen to be barely affected (Figure 3C). Hydrogen sulfide and methanethiol show no signs of significant change above 0.5% ethanol (Figure 3D). These phenomena may be an indication of a more adsorptive mechanism, while the Carboxen allows a more absorptive mechanism [9]. This is due to the uniform structure of solid microspheres within the DVB phase, compared to the non-uniform porosity of activated charcoal.

### 2.2. Ratio Analysis

It was shown that different sulfur compounds were not affected by ethanol concentration in the same way. This causes an issue with wine analysis, where two different wines could have up to a 20–30% difference in ethanol concentration, or even more so if brandies or other spirits are involved. However, calibration and quantification rely on the ratio of analytes to internal standards. If an internal standard is selected that most closely mimics the behavior of an analyte in question, its ratio, at constant concentrations of both, will be constant despite an increase in ethanol. Thus, proper internal standards are necessary for accurate quantification.

EMS and DIDS have been used as internal standards to measure smaller (DMS, thioacetates, etc.) and larger (DEDS, DMTS) compounds respectively [8,14], though other compounds have been used, including hexylmercaptan, propylthioacetate [11], 4-methylthiobutanol [8], thiophene [15], etc. In addition to the traditional EMS and DIDS, this study used the internal standards DES, MIS, and EIS. These sulfides were selected for the protective nature of their large substituents, in an effort to reduce the outside influence on the central sulfur atom. Diisopropyl sulfide (DIS) was attempted to be used, however, it co-eluted with ethanol, causing an irregular peak due to quenching effects.

**Figure 4:**
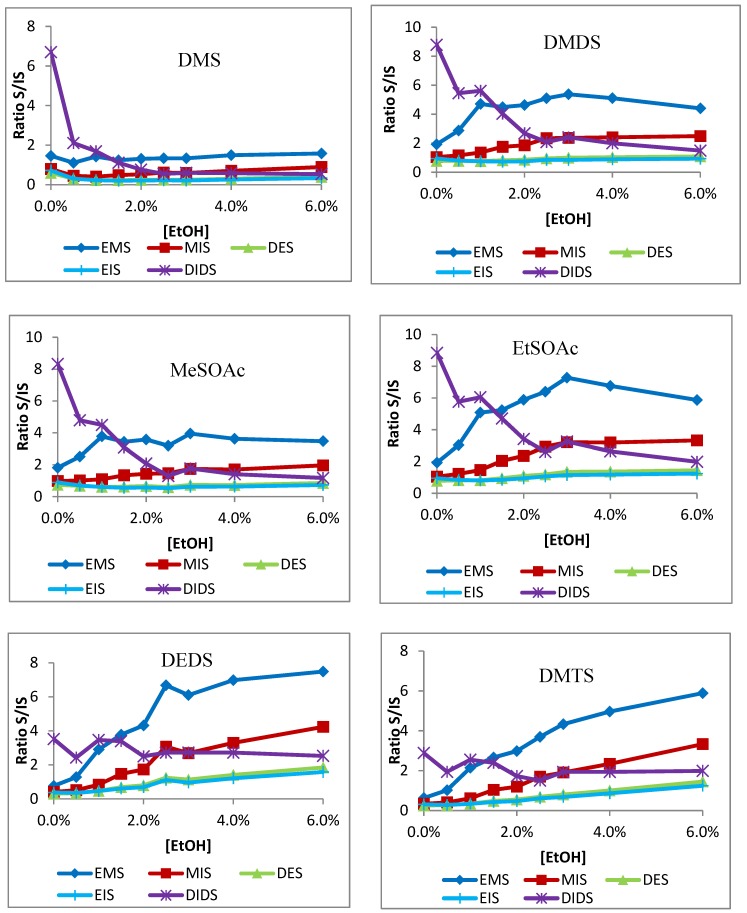

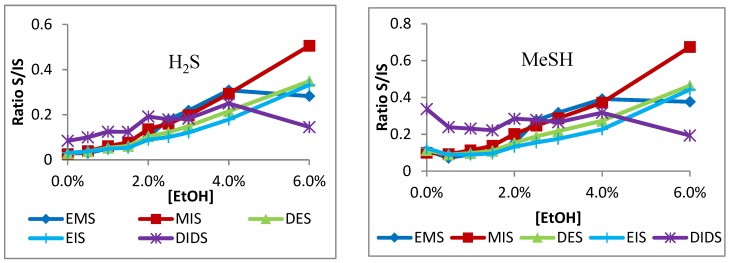
Analyte to internal standard ratios (based on square roots of peak areas) to each of the five internal standards of DMS, DMDS, MeSOAc, EtSOAc, DEDS, DMTS, H_2_S, and MeSH on Carboxen/PDMS fiber.

The results from the analyte-to-internal-standard ratio tests on Carboxen-PDMS fiber are shown in Figure 4. As the Carboxen-PDMS fiber had the greatest sensitivity, and the ratios were at least as consistent as the other fibers, it was decided to only pursue a single fiber for analysis. DMS is most closely matched by EMS, as indicated by a flat curve shape throughout the increase of ethanol concentration. However, all other analytes show a large discrepancy when compared to EMS. The data suggest that DMDS and the thioacetates may be better suited with DES or EIS. DEDS and DMTS seem to most closely follow DIDS. H_2_S and MeSH, counter-intuitively, seem to follow DIDS, rather than EMS, as might be expected from their size. The direct analysis curves of H_2_S, MeSH, and DIDS show a similar gradual decrease with rising ethanol concentration, suggesting a less extreme influence. This may be a function of molecular size and displacement on the SPME fiber. For instance, DIDS is a relatively large compound that could displace smaller compounds, and H_2_S and MeSH are small enough to fit in the minute pores of the Carboxen phase and avoid displacement. This could decrease the effect of ethanol competition for both, as more mid-sized compounds may lack the ability to displace ethanol or remain on the fiber against larger compounds. Furthermore, between 0.0% and 1.0% ethanol, all ratios are unstable. This suggests a minimum of 1.0% ethanol is required for accuracy, above which the ratios become more consistent.

Similar effects were seen using the DVB-Carboxen-PDMS and DVB-PDMS fibers (data not shown). However, because the sensitivity was greatest on the traditional Carboxen-PDMS fiber, and as the ratio analysis suggests accurate measurements within defined ethanol ranges, it was decided to pursue the single fiber for further analysis. Based on the apparent stabilization above 1% EtOH, the ideal wine sample would be diluted to 2 mL wine:8 mL saturated salt water. Wine commonly ranges between 12–15% ethanol, which, after dilution, is reduced to 2.4–3%. It is within this range that analyte to internal standard ratios are best maintained, especially in the case of H_2_S and MeSH, in which a consistent correlation was not found across the entire ethanol range. Thus, in order to avoid strong ethanol matrix effects, internal standards EMS, EIS, and DIDS should be used on Carboxen-PDMS fiber. DMS should be measured against EMS; DMDS, MeSOAc, and EtSOAc should be measured against EIS, and DEDS, DMTS, H_2_S, and MeSH should be measured against DIDS.

## 3. Materials and Methods

### 3.1. Chemicals

Sodium sulfide, methanethiol (MeSH), dimethyl disulfide (DMDS), dimethyl trisulfide (DMTS), and diisopropyl disulfide (DIDS) were obtained from Sigma-Aldrich (St. Louis, MO, USA). Methyl thioacetate (MeSOAc), ethyl thioacetate (EtSOAc), and diethyl sulfide (DES) were obtained from Alfa-Aesar (Ward Hill, MA, USA). Ethyl methyl sulfide (EMS), dimethyl sulfide (DMS), diethyl disulfide (DEDS), methyl isopropyl sulfide (MIS), and ethyl isopropyl sulfide (EIS) were obtained from TCI America (Portland, OR, USA). Methanol was obtained from EMD Chemicals Inc. (Gibbstown, NJ, USA), l-tartaric acid was obtained from J.T. Baker (Phillipsburg, NJ, USA), and ethanol was obtained from Koptec (King of Prussia, PA, USA).

### 3.2. Sample Preparation

Hydrogen sulfide standards were prepared using equivalents of sodium sulfide (Na_2_S) dissolved in distilled water and further diluted with cold (−15 °C) methanol. MeSH standards were prepared by slowly bubbling the pure gas over cold methanol and recording the gained mass. All other standards were prepared by dilution with cold methanol. A standard mixture (mix 1) was prepared, containing DMS (3000 μg/L), MeSOAc (1285 µg/L), DMDS (218 µg/L), EtSOAc (564 µg/L), DEDS (55 μg/L), and DMTS (47 µg/L). Because MeSH readily oxidizes to DMDS, and the higher affinity for DMDS on the SPME fiber causes much greater peak responses, the two compounds were not analyzed simultaneously. A separate mixture (mix 2) was thus prepared, containing MeSH (36.9 mg/L) and H_2_S (31.25 µg/L). Finally, a mixture of internal standards (IS mix) was prepared containing EMS (5 mg/L), DES (1 mg/L), MIS (1.5 mg/L), EIS (1 mg/L), and DIDS (25.9 µg/L).

Samples were prepared in 20 mL deactivated screw-cap glass vials with Teflon-faced silicone septa. Synthetic wine was prepared using a 3.5 g/L tartaric acid solution in 12% ethanol, and 2 mL of synthetic wine was used for analysis. Ethanol was added corresponding to target concentrations of 0.0, 0.5, 1.0, 1.5, 2.0, 2.5, 3.0, 4.0, and 6.0%. Saturated salt water was then added to a total volume of 10 mL in order to drive more volatiles into the headspace [9]. The vials were flushed gently with argon under a low flow rate (barely disturbing the surface of the sample liquid) to avoid turbulence. All samples received 20 µL of the standard mixture (mix 1 or 2) and 10 µL of the IS mix (thus 30 µL of methanolic solutions added in total). In the case of mix 2, all standards were introduced via a syringe through the sample-vial septum to avoid oxygen contact. All standards were stored in a freezer (−15 °C).

### 3.3. SPME Conditions

Three SPME fibers were used: 85 µm Carboxen-PDMS, 65 µm poly(divinylbenzene)(DVB)-PDMS, and 50/30 µm DVB-Carboxen-PDMS (Supelco, Bellafonte, PA, USA). The samples were equilibrated at 30 °C for 5 min, and the extraction took place for 20 min with agitation at 250 rpm. The injection temperatures for each fiber were 300 °C, 250 °C, and 270 °C, respectively. The samples were analyzed in triplicate.

### 3.4. GC-PFPD

The samples were run on a Varian CP-3800 gas chromatograph equipped with a pulsed-flame photometric detector (PFPD; Varian, Walnut Creek, CA, USA). A DB-FFAP column (30 m × 0.32 mm × 1 µm, Agilent, Palo Alto, CA, USA) was used for separation. A temperature program was used for the GC oven: 35 °C for 3 min, ramped to 150 °C at 10 °C/min, held 5 min, ramped to 220 °C at 20 °C/min, held 3 min. Nitrogen was used as the carrier gas at 2 mL/min flow rate. The detector temperature was 300 °C with 14 mL/min hydrogen, 17 mL/min air 1, and 10 mL/min air 2. The PFPD was operating in sulfur mode, with a 6 ms gate delay and a 20 ms gate width. Data analysis relied on the square roots of peak areas.

## 4. Conclusions

In order to accurately analyze volatile sulfur compounds, despite matrix parameters, a suitable internal standard must be found that will be affected in the same manner as the analyte of interest. Three fibers and five internal standards were used to analyze volatile sulfur compounds in synthetic wine matrices in order to determine the best method to compensate for said effects. The Carboxen-PDMS fiber showed the greatest sensitivity compared to the other analyzed fibers. The effects of ethanol on sulfur analysis were very substantial, for instance, a great decrease in sensitivity was seen between 0.0 and 0.5%. The ratios of analyte to internal standard were in most levels between 2–4% ethanol, suggesting a dilution of 2 mL wine to 8 mL salt water is satisfactory for analysis. Both H_2_S and MeSH, traditionally analyzed with EMS as an internal standard, showed poor resemblance to EMS with increasing ethanol content. DIDS was found to be a more suitable internal standard, despite their dissimilarities, based on their response to the matrix and their behavior on the fiber.
